# The Surgical Aid Society

**Published:** 1904-12-10

**Authors:** 


### THE SURGICAL AID SOCIETY.

### SIR F. TREVES AT THE MANSION HOUSE.

THE forty-second annual meeting of the Surgical Aid Society was held at the Mansion House on Monday, 5th inst., the Lord Mayor presiding. There was a good attendance, many ladies being present.

The report showed that while the past year had been an uneventful one, it had again witnessed a most encouraging advance in income, as well as in the amount of practical work accomplished. His Majesty the King had again doubled his annual subscription, and the total amount of subscriptions showed an increase of £740 17s. 8d., while life subscriptions had advanced by £401 11s. The net income, including legacies, was £26,937, representing an increase of £3,501 11s. 4d. on that of last year. This was mainly attributable to a legacy of £8,198, 18s. under the will of the late Miss May Thompson, but even eliminating this source of income, the total for the year was £18,506 7s. 10d., compared with £17,520 2s. 8d. received in 1903. The expenditure in actual relief and suffering was £1,415 2s. 8d. more than the amount thus expended in the previous year, being £16,636 8s. 11d. The expenditure for relief stood to that of management in the ratio of 83·44 per cent. to 16·56; or, in other words, rather more than 16s. 8d. in every £1 went in actual relief and suffering.

The Lord Mayor, in testifying to the valuable work accomplished by the society, drew attention to the fact that since its formation in 1862, 304,272 patients had been relieved, and 463,676 appliances supplied. Among the latest benefits conferred by it was the supply of an artificial hand to the fisherman Hogarth, who was wounded in the late regrettable incident in the North Sea. While expressing the deep regret of the society occasioned by the death of Mr. H. W. Allingham, hon. consulting surgeon to the society, he welcomed Sir Frederick Treves as a worthy successor.

Mr. Sheriff G. J. Woodman, J.P., moved a resolution to the effect that, while rejoicing in the success of the past year, the meeting pledged itself to use every effort to extend the usefulness of the society and augment its resources. During a particularly bad year, when most charitable enterprises suffered severely, this institution had not only maintained its position, but increased its subscriptions, and could show a larger income than ever before, thus testifying to the estimation in which it was held by the general public. Not only had his Majesty doubled his subscription, but the Livery Companies of London had maintained, and in some instances increased, their contributions. The number of appliances had also been increased, 21,304 sufferers having been relieved—an average of 410 per week—of whom by far the largest proportion were women and children. The number of appliances supplied in the 12 months was 34,411, as compared with 32,264 in the previous year. He called special attention to the fact that the society gave the privileges of tickets even to those subscribers who could only afford half a guinea, and that there was not, as in some cases, a time-limit to these tickets. He thought this very desirable, and that more attention might be drawn to it in the publications of the society. On the seconding of Mr. C. Turner Room, the resolution was carried, and the report and accounts were adopted.

Sir Horace Brooks Marshall having spoken in sympathetic and highly appreciative terms of the late consulting surgeon, Mr. Allingham, Sir Frederick Treves was unanimously elected to fill the vacancy.

Sir F. Treves, in proposing a vote of thanks to the surgeons connected with the Society, said a society of this kind required skilled surgeons, and, as everyone knew, expert surgical and medical advice was necessarily costly. He found, however, that these three surgeons, well known as they were throughout the whole length and breadth of the surgical world, as peculiarly eminent and qualified to carry on the particular work of this society—the best men, so far as he knew, that the city of London could supply—seeing between them no less than 410 patients per week, were receiving emoluments at the rate of only 15s. per visit. He did not think there could be any complaint of extravagance on that score. No stronger proof of the high esteem felt for the society by the profession could, he thought, be required, than the fact that these men were practically giving their services for nothing. At first sight, no doubt, the society's work did not appeal to the charitable public. No one could speak more warmly than he of the magnificent work being done by the hospitals, but while the hospitals did their best to get a patient well, the Surgical Aid Society kept him well, and without it a large amount of the work of the metropolitan and provincial hospitals would be more or less valueless. As an illustration, he would remark that an operation for cataract would be useless without the provision of suitable glasses; thus a large number of operations left a man scientifically well, but unless he were secured against relapse, and enabled to follow an occupation, he was not in a position to benefit by the treatment he had undergone. It was for this reason that he specially desired to bring the excellent work of the society to the notice of employers of labour, to whose interest it was to secure the highest working efficiency of their employees, and their speedy return, after accident, to their position as wage-earners. The society being a sort of supply association, he would suggest that, Christmas being near, when vendors of wares set about displaying them in as attractive a form as possible, the society might draw attention very graphically to its labours by setting out in the form of an exhibition the various surgical appliances which, he saw from the report, had been supplied by it during the year. From his long connection with hospitals, he could say emphatically that the work of this society was absolutely indispensable. While it was not showy, and did not appeal at once to the romance and sentiment of charity, it was one of the soundest, most substantial, and most necessary works carried on in this great city.

The Rev. O. Ensor Walters, of the West London Mission, having seconded, the resolution was carried, and Mr. E. Muirhead Little responded on behalf of the surgical staff.

Mr. G. H. Ryan moved that a cordial vote of thanks be given to Mr. Samuel Watson, treasurer, and that he be asked to continue in office. Mr. Watson was, he remarked, indefatigable in the attention he gave to the business of the society, and combined a rare power of discrimination with much human sympathy.

The re-election of the treasurer having been effected, the Rev. W. C. Hawkesley, in proposing a vote of thanks to the committee, and their re-election, emphasised the value of the work of the society in the large provincial towns; and Sir W. Treloar urged the claims of the society as one of the most worthy of support in the City of London.

The amount realised in connection with the meeting was announced by the secretary as £449 6s. 6d.; and a vote of thanks to the Lord Mayor brought the proceedings to a close.

